# OnabotulinumtoxinA in the Management of Pain in Adult Patients with Spasticity: A Systematic Literature Review

**DOI:** 10.3390/toxins17080418

**Published:** 2025-08-18

**Authors:** Francesca Bianchi, Mariana Nelson, Jörg Wissel, Heakyung Kim, Alexandra Traut, Darshini Shah, Tiziana Musacchio, Bahman Jabbari

**Affiliations:** 1 Neurology and Stroke Unit, Department of Neuroscience, Bufalini Hospital, 47521 Cesena, Italy; francesca.bianchi@auslromagna.it; 2 AbbVie, 1 North Waukegan Road, North Chicago, IL 60064, USA; mariana.nelson@abbvie.com (M.N.); alexandra.traut@abbvie.com (A.T.); darshini.shah@abbvie.com (D.S.); 3 Neurology and Psychosomatic at Wittenbergplatz, University of Potsdam, 14469 Potsdam, Germany; joerg@schwarz-wissel.de; 4 UT Southwestern Medical Center, Dallas, TX 75390, USA; heakyung.kim@utsouthwestern.edu; 5 Department of Neurology, Yale University, New Haven, CT 06510, USA; bahman.jabbari@yale.edu

**Keywords:** botulinum toxin A, BOTOX, lower limb, multiple sclerosis, pain, post-stroke, post-traumatic, SLR, spasticity, upper limb

## Abstract

Many patients with spasticity report pain which can be debilitating. Numerous studies have shown onabotulinumtoxinA (onabotA) is efficacious in the management of spasticity but comprehensive data on its impact on spasticity-associated pain is limited. This systematic review aimed to assess the published evidence on the efficacy of onabotA in the management of pain in adults with spasticity. Search strategies were conducted from 1990 to 2023 for journal publications and from 2020 to 2023 for congress proceedings to identify relevant studies on onabotA in adults with spasticity where pain was a reported outcome. Of 665 records identified, 31 unique studies from 33 publications were included (2740 patients). Twenty-seven studies demonstrated a reduction in pain compared to baseline following treatment with onabotA in adults with spasticity (n = 2740). Of these, 12 studies reported a statistically significant reduction in pain with onabotA versus baseline. Sixteen studies reported a clinically meaningful reduction in pain (≥30% reduction). The reduction in pain with onabotA was consistent across etiologies and a range of pain measures. There was a high level of heterogeneity in the design and quality of the studies identified, which limited statistical analysis; however, the published evidence overall shows a consistent positive trend for the use of onabotA in reducing spasticity-related pain in adults.

## 1. Introduction

Spasticity is a motor control disorder that is part of the upper motor neuron syndrome, characterized by muscle stiffness, tightness, and involuntary muscle contractions, which can lead to difficulties in movement and coordination [1,2]. It can result from a single insult event such as traumatic brain injury (TBI), spinal cord injury (SCI), stroke, or cerebral palsy (CP), as well as many chronic neurological conditions such as multiple sclerosis, hereditary spastic paraparesis, or motor neuron disease (MND) [3,4]. Patients with spasticity frequently experience pain [5], the prevalence of which varies between neurological conditions. Studies have reported pain in 75% of adults with CP, 65% of patients with upper motor neuron lesions, 30–60% of patients with MS and SCI, 39% of patients with chronic stroke, and 15% of patients with acute stroke [6,7,8,9,10,11]. Although the pathophysiology of spasticity-related pain is not fully understood, the combination of neurogenic factors and biomechanical changes associated with spasticity are believed to underlie the symptoms [12]. Pain may be associated with muscular hyperactivity and spasms due to ischemia resulting from compression of the muscle blood vessels leading to activation of muscle nociceptors [13]. Alternatively, secondary peripheral structural changes (e.g., muscle and tendon shortening and contractures) may lead to aberrant biomechanical forces on the limbs and trunk, causing pain due to structural misalignment of the joints and skeleton [12]. Pain in patients with spasticity has the potential to severely compromise patients’ quality of life (QoL) by interfering with daily tasks, disturbing sleep, and deteriorating mental and physical health [14]. In terms of managing pain related to spasticity, there is limited evidence that existing oral therapies adequately address pain symptoms [15], and many interventions are not licensed specifically for the alleviation of pain associated with spasticity [16]; thus, this aspect remains a challenge.

Botulinum toxin type A (BoNT/A) is an effective treatment option for the management of adult spasticity [17] and onabotulinumtoxinA (onabotA) is approved for treatment of upper and lower spasticity in both pediatric and adult patients. OnabotA emerged as a recommended treatment of choice in patients with spasticity due to its well-established safety, long-term tolerability, and efficacy across a range of doses. It has also been associated with improvement in symptoms and QoL, high patient and physician satisfaction, and the potential to reduce costs and healthcare utilization [18,19,20,21]. OnabotA has been shown to improve symptoms associated with spasticity such as pain [22], although the mechanism of action is not fully understood. Furthermore, onabotA has demonstrated efficacy in reducing pain in other medical indications not associated with spasticity, such as prophylaxis of headaches in adults with chronic migraine (CM) [23,24] and in patients with cervical dystonia (CD) [25,26,27], through multiple mechanisms [28,29]. Although the antinociceptive effects of BoNT/A were initially attributed to its myorelaxant action, there is ample preclinical evidence that peripheral and central sensitization is counteracted by BoNT/A [29]. Mechanisms proposed to play a role in the antinociceptive action of BoNT/A include inhibition of neurotransmitters release from nerve terminals resulting in reduced peripheral sensitization, increased Schwann cell proliferation with possible regenerative effect after nerve injury, prevention of upregulation of pain-related ion channels and inhibition of satellite glial cells activation in dorsal root ganglia, prevention of central neurotransmitter release and microglia activation, and modulation of spinal opioidergic and GABAergic system resulting in reduced central sensitization [29,30].

Currently, three major BoNT/A formulations onabotA (BOTOX^®^, AbbVie, North Chicago, IL, United States and Allergan, Dublin, Leinster, Republic of Ireland), abobotulinumtoxinA (Dysport^®^, Ipsen, Paris, France), and incobotulinumtoxinA (Xeomin^®^, Merz, Raleigh, NC, USA) [31,32,33] are approved across different indications [34,35,36]. These formulations have unique physiochemical characteristics that result in distinct product profiles [37]. BoNT/A formulations also have different assays used to measure unit potency and are thus not interchangeable [37].

A systematic literature review (SLR) conducted in 2021 evaluated the clinical effectiveness, safety, and humanistic and economic impact of onabotA in treating upper and/or lower limb spasticity in adults. This broad SLR identified 78 publications that demonstrated significantly improved muscle tone and functional disability in patients with spasticity following treatment with onabotA. In an exploratory analysis of the data, many studies showed statistical or clinically meaningful reductions in pain following administration of onabotA in adults with upper and lower limb spasticity. However, the SLR did not focus on pain as an outcome and thus did not extract all pain-related relevant endpoints. A further 2021 SLR and meta-analysis was conducted to assess BoNT/A in post-stroke upper limb spasticity; however, this focused on the effectiveness of BoNT/A in combination with constraint-induced movement therapy and also did not report pain as an outcome [38]. Therefore, the current SLR was conducted to specifically assess the evidence on the efficacy of onabotA in pain as an outcome in adults with spasticity of any etiology.

## 2. Results

A total of 665 articles were identified using the defined search strategy. Title and abstract screening identified 158 full-text articles meeting the eligibility criteria which were defined in the Population, Intervention, Comparator, Outcomes, Study design-Timeframe (PICOS-T) (Table 1). Publications were only included if they assessed pain as an outcome and included onabotA for the treatment of spasticity.

**Figure 1:**
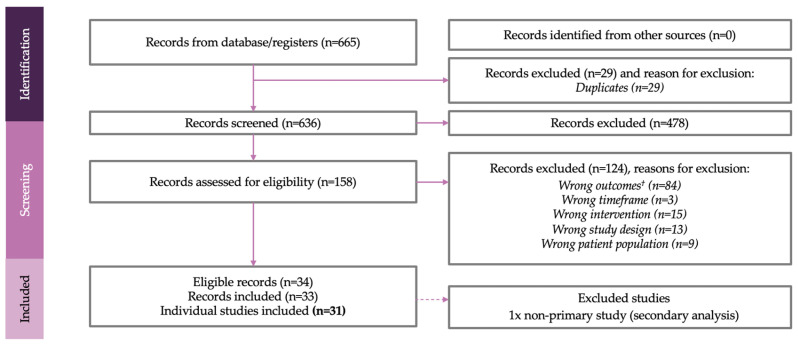
PRISMA flow diagram. ^†^ Wrong outcomes relate to studies not examining pain outcomes after treatment with onabotA.

Following full-text screening, 124 articles were excluded. Thirty-four articles were included in the final analysis. One study was excluded during data extraction as it was not a primary study, resulting in 33 included articles. Results from one study were reported across two separate publications; therefore, 31 unique studies from 33 publications were included in this review (Figure 1).

Details of the publications included in this SLR are presented in Table 2.

### 2.1. Study Characteristics

The SLR included 14 randomized controlled trials (RCTs) [14,40,41,43,48,49,50,51,52,53,54,62,63,69,70] and 17 observational studies [39,42,44,45,46,47,55,56,57,58,59,60,61,64,65,66,67,68]. All observational studies were cohort studies, with the majority (n = 13) assessing a single overall cohort. Of the remainder, one study compared the impact of onabotA on patients with acute or chronic spasticity [68]; one study compared onabotA in patients with varying spastic etiologies [42]; one study assessed patients treated with onabotA or abobotulinumtoxinA [56]; and one study compared onabotA in treatment-naïve patients to those on maintenance therapy [47]. There were no economic analyses identified in this review.

**Figure 2:**
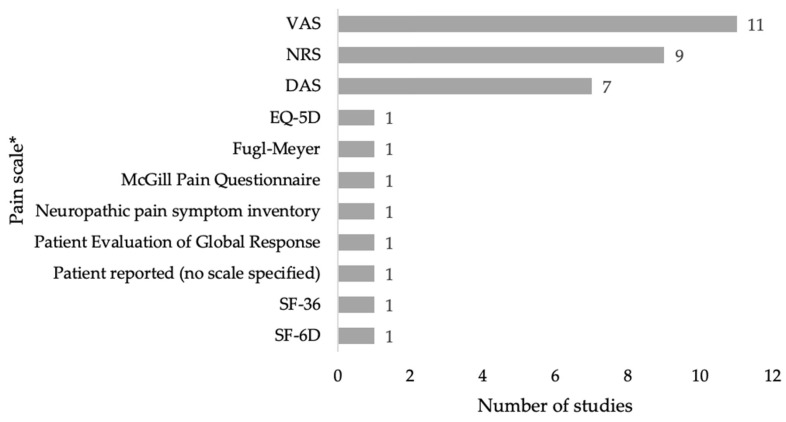
Scale used to assess pain outcomes of included studies. * Several papers included more than one scale and have been counted separately. DAS, Disability Assessment Scale; EQ-5D, EuroQoL 5-Dimension; NRS, numerical rating scale; SF-36, short-form 36-items survey; SF-6D, short-form six-dimension; VAS, visual analog scale.

Most studies were conducted in Europe (n = 14) [39,41,42,53,54,56,57,58,59,60,61,64,68,70], with the remaining in the United States (n = 6) [14,40,43,46,55,63], Asia (n = 7) [48,51,52,62,65,66,67], and Australia (n = 1) [49,50]. Two studies reported in three publications were conducted across differing geographies [44,45,69]. In all studies, the measurement of pain was reported by the patient and was thus subjective. The majority of studies utilized the visual analog scale (VAS) (n = 11) [14,39,41,49,50,54,56,57,60,64,65,66] or a numerical rating scale (NRS) (n = 9) [40,42,44,45,52,58,59,61,67,69] but standardized assessments such as the disability assessment scale (DAS) (n = 8) [14,44,45,46,48,51,62,70], EuroQoL 5-Dimension (EQ-5D) (n = 1) [70], Fugl-Meyer (n = 1) [43], short-form 6-dimension (SF-6D) (n = 1) [47], and short-form-36 (SF-36) (n = 1) [53], all of which include pain measurements as a sub-domain, were also included (Figure 2).

### 2.2. Study Population

Across all studies, 3225 patients received any formulation of BoNT/A. When focusing only on patients who received onabotA, 2740 patients were included in this SLR (485 patients who received other formulations were excluded). The cause of spasticity was documented in all publications, with stroke being the most common etiology (67.5%, n = 2177). Other reasons for spasticity included CP (7.3%, n = 236), MS (5.2%, n = 170), SCI (3.0%, n = 98), TBI (2.8%, n = 92), head injury (0.24%, n = 8), transverse myelitis (0.12%, n = 4), and hypoxic brain injury (0.06%, n = 2). The type of stroke was reported in nine studies (n = 503), with ischemic stroke being the most common (66.8%, n = 336). In studies that reported location of spasticity, 62% of subjects presented with upper limb spasticity (n = 1601), 32% with lower limb spasticity (n = 834), and 6% with both (n = 151). The location of pain was reported in six studies: three studies examined patients with shoulder pain; one study examined wrist pain; and two studies examined pain from a range of locations, including foot, shoulder, elbow, wrist, and fingers.

### 2.3. Treatment Characteristics

Mean overall onabotA dosage was reported in 24 studies and ranged from 75 U to 540 U [54,63]. Most studies reported a single dose of onabotA treatment; however, six studies reported multiple treatments (two studies reported up to 2 treatment cycles [40,69], one study reported a mean of 2.8 treatments [46], one study reported 4 doses [70], and the ASPIRE study reported 8 doses of treatment [44,45]). Twenty-six studies reported which muscles were injected and this was generally at the discretion of the clinician and directed by the location of spasticity. Fourteen studies reported adjunct pharmaceutical and non-pharmaceutical treatments, which included physical therapy/physiotherapy (n = 982) among other training exercises and orthopedic resources.

### 2.4. Clinical Efficacy

All studies that reported a pain outcome were included, regardless of the scale used. The VAS, NRS, and DAS were the most commonly used instruments in eleven, nine, and seven studies, respectively. Six additional pain scales (EQ-5D, SF-36, SF-6D, the McGill pain questionnaire [MPQ], Neuropathic Pain Symptom Inventory [NPSI], and Fugl-Meyer) were identified, but these were used infrequently.

#### 2.4.1. Visual Analog Scale

**Figure 3:**
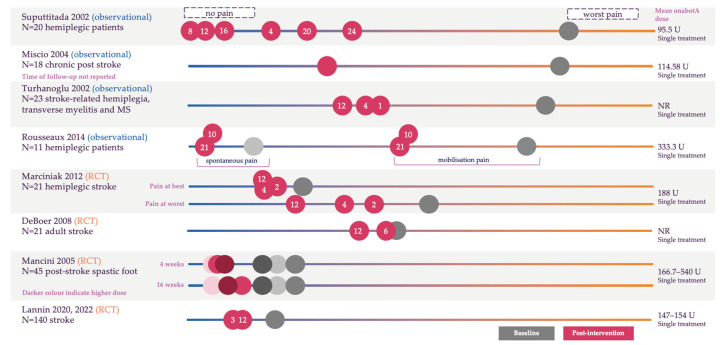
Change in visual analog scale from baseline following treatment with onabotA [14,41,49,50,54,56,60,65,66]. Please note this is a visual representation of the data and is not to scale; for the full dataset, please see Supplementary Materials Table S4. The numbers in circles indicate the weeks of follow-up, with grey circles indicating baseline score and red circles indicating post-onabotA treatment. In Mancini 2005, darker shading indicates higher dose. Scales represent either 0–10 or 0–100 (no pain to worst pain). HTM, hemiplegia transverse myelitis; MS, multiple sclerosis; NR, not reported; RCT, randomized controlled trial; SCI, spinal cord injury; U, units.

Eleven studies measured pain using a VAS [14,39,41,49,50,54,56,57,60,64,65,66]; in ten of these studies, pain decreased from baseline following treatment with onabotA during a follow-up period of between 1 and 52 weeks. In one study, there was a very high response rate to onabotA treatment, with 94% of patients reporting a reduction in pain at 6 weeks [39]. Detailed VAS data were not reported in three studies; the findings from the remaining eight studies are presented in Figure 3.

Three studies reported a statistically significant reduction 3–5 months after a single treatment across a range of different etiologies [54,60,66]. One study examined two cohorts of patients who received onabotA following a stroke for moderate to severe spasticity of the wrist and were administered onabotA with evidence-based movement training or onabotA with a handout of rehabilitation exercises. Patients showed a statistically significant improvement in pain when the two cohorts who received onabotA were combined, although the change from baseline in the two separate cohorts was not statistically significant [49].

#### 2.4.2. Numerical Rating Scale

**Figure 4:**
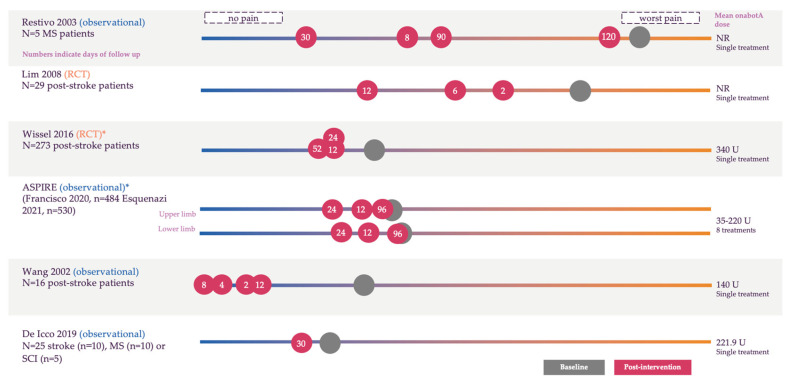
Change in numerical rating scale from baseline following treatment with onabotA [42,44,45,52,58,67,69]. Please note this is a visual representation of the data and is not to scale; for the full dataset, please see Supplementary Materials Table S5. The numbers in circles indicate the weeks of follow-up, with grey circles indicating baseline score and red circles indicating post-onabotA treatment. Numbers in the circles indicate the weeks of follow-up. Scales were 0–3 [58] or 0–10 (all other studies). ASPIRE, Adult SPasticity International Registry; MS, multiple sclerosis; NR, not reported; RCT, randomized controlled trial; SCI, spinal cord injury; U, units. * Post-treatment values were calculated based on average reduction.

Nine studies from 10 publications measured pain with an NRS using scales ranging from 0–3 to 0–10 points [40,42,44,45,52,58,59,61,67,69]. Of these 10 publications, Sampaoi et al. 1997 only reported pain in two patients, Childers et al. did not report post-treatment pain scores, and Rousseaux et al. reported very low mean pain scores on a 0–6 scale (0.31 at baseline and 0.24, 0.28, and 0.19 at 15 days, 2 months, and 5 months, respectively); thus, the results from the remaining seven publications are visualized in Figure 4.

Eight of the nine studies showed a reduction in pain post-onabotA treatment compared to baseline and, of these, five showed a statistically significant reduction [44,45,58,67,69]. Two studies did not report whether the results were statistically significant [40,59] and four studies reported statistical significance only at specific time points [45,52,58,69].

A randomized, double-blind, placebo-controlled, phase IIIb study [69] in 273 patients with post-stroke spasticity showed a significant reduction in pain following up to two treatment cycles with onabotA that was maintained up to 52 weeks compared to placebo. Patients in the onabotA cohort experienced greater average changes (95% CI) from baseline compared to the placebo cohort at weeks 12 (−0.77 [1.14–0.40] vs. −0.13 [0.51–0.24], *p* = 0.019), 24 (−0.78 [1.22–0.34] vs. −0.13 [0.58–0.31], *p* = 0.043), and 52 (−1.08 [1.52–0.65] vs. 0.67 [1.12–0.22], *p*-value not reported) [69].

The only comparative study in stroke patients was a randomized, double-blind, controlled trial that compared onabotA (n = 16) and triamcinolone acetonide (n = 13) for hemiplegic shoulder pain in 29 post-stroke patients up to 12 weeks [52]. Pain scores at weeks 2, 6, and 12 were 5.9, 6.0, and 4.9 for onabotA and 5.5, 3.2, and 5.2 for triamcinolone acetonide (*p* = 0.064). At week 12, an intention-to-treat analysis was also conducted. Mean baseline scores were 7.9 for onabotA and 7.6 for triamcinolone acetonide, which decreased to 4.2 in the onabotA group and 2.5 in the triamcinolone acetonide group (*p* = 0.051) [52].

#### 2.4.3. Disability Assessment Scale

The DAS was used in seven studies (eight publications) [14,44,45,46,48,51,62,70]. One study is not included in the analysis as details of the pain-associated DAS score were not documented [48]. Four of the remaining six studies showed a reduction in pain post-onabotA versus baseline [44,45,46,51,70], and three studies reported statistically significant reductions [44,45,51].

**Figure 5:**
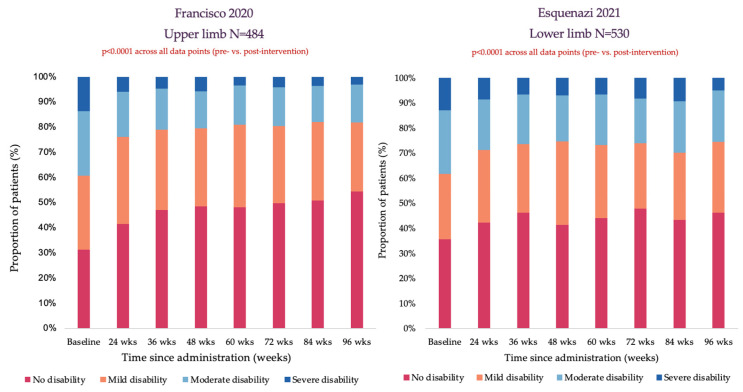
Pain measured by the DAS was significantly reduced after onabotA treatment at all time points in patients with upper limb and lower limb spasticity in the ASPIRE study. ASPIRE, The Adult Spasticity International Registry; DAS, disability assessment scale; wks, weeks.

The ASPIRE study, a multicenter, prospective, observational study, assessed the real-world utilization of onabotA and its impact on spasticity-associated pain. As well as using an 11-point NRS, pain was assessed using the DAS, where patients were evaluated by clinicians over a follow-up period of 96 weeks. Study results describing pain outcomes were reported in two different publications in this systematic review. Francisco et al. 2020 reported results in patients with upper limb spasticity (n = 484) [45], and Esquenazi et al. 2021 reported results in patients with lower limb spasticity (n = 530, Figure 5) [44]. A similar trend of decreased severe pain-related disability and increased reporting of no disability was observed in both publications across the study period. Furthermore, both publications reported a significant reduction in patient-reported spasticity-related pain evaluated with NRS (as reported in 4.2.2).

Two studies compared onabotA with an active comparator using the DAS [51,62]. One multicenter, randomized, controlled trial [62] evaluated Neuronox^®^ compared to onabotA for the treatment of post-stroke upper limb spasticity (n = 196). There was no significant improvement in pain-associated DAS at week 4, 8, or 12 compared to baseline. Additionally, there was no significant difference between the Neuronox^®^ and onabotA groups in the changes in DAS from baseline; the study did not provide any possible reason for these effects [62].

A prospective, randomized, double-blind study compared onabotA to Coretox^®^ (a 150 kDa complexing protein-free BOTN/A formulation, Medytox, Seoul, South Korea) in 220 patients with post-stroke upper limb spasticity [51]. Participants received either a single treatment of Coretox (100U) or onabotA (100 U). OnabotA-treated individuals experienced a significant decrease in mean (SD) DAS pain score compared to baseline at 4 weeks (−0.86 [0.69], *p* = 0.0167), 8 weeks (−1.43 [0.53], *p* = 0.0156), and 12 weeks (−1.43 [0.53], *p* = 0.0156). There was no significant difference in DAS score between onabotA and Coretox treatment groups at all time points (*p* > 0.05) [51]. It should be noted that all BoNT/A formulations have unique physiochemical characteristics and are not interchangeable due to differences introduced at each step of the manufacturing process [37]; thus, formal comparison between formulations cannot be made.

#### 2.4.4. Additional Measures of Pain

Other scales for the measurement of pain, including the MPQ, the pain/discomfort item of the EQ-5D, and bodily pain sub-domains of the SF-36 and SF-6D, were used in nine studies [14,42,43,47,53,55,63,68,70]. All studies demonstrated a reduction in pain following treatment with onabotA compared to baseline, with two studies reporting statistically significant reductions in spasticity-associated pain [53,68].

#### 2.4.5. Clinically Meaningful Reductions in Pain

In addition to statistical significance, this SLR examined studies for clinically meaningful reductions in pain. The literature defines a 30% reduction in pain from baseline as clinically meaningful and this was used to analyze the percentage change in pain [71]. Only studies that reported mean or median baseline and post-intervention pain values were included in this analysis. Overall, 16 studies (17 publications) out of 31 demonstrated a ≥30% reduction in pain with onabotA compared to baseline at some or all time points, ranging from 1 to 84 weeks [14,42,43,47,48,49,50,52,54,56,57,58,63,64,65,66,67]. Four weeks was the most common follow-up time when a clinically meaningful reduction in pain was demonstrated, and this was reported in seven studies [14,51,54,58,65,66,67]. Additionally, ten studies reported a reduction in pain of ≥50% and five studies reported a reduction in pain of ≥70% compared to baseline (no additional analyses were performed on these groups since the analysis primarily focused on the >30% reduction cut-off).

### 2.5. Risk of Bias

All RCTs were analyzed for potential risk of bias using the Cochrane Risk of Bias Tool v2.0 (Table 3). Judgement about the risk of bias for the five set domains of the Cochrane tool used the Cochrane algorithm to determine whether the risk was ‘low’, ‘high’, or raised ‘some concerns’. In total, 6 of 14 RCTs were considered high-quality with a low risk of bias [48,51,52,53,62,69]. Five studies were judged to raise some concerns in their risk of bias [14,40,41,54,63]. These were attributable to lack of information on the patient randomization process [41], no information on the number of participants completing the study period [54], no pre-specified analysis plan [14,40,54], or no information on whether the selection of results arose from multiple analyses of the data [63]. Three studies (four publications) were judged to be at high risk of bias due to the likelihood that the self-reported pain assessment was influenced by patient knowledge of their assigned study cohort [43,49,50,70].

The Newcastle–Ottawa Scale was used to assess the quality of non-randomized studies. A ‘star system’ is used to judge studies on three broad perspectives: the selection of the study groups; the comparability of the groups; and the ascertainment of the outcome of interest. Each item is graded one point, except for comparability, which can be scored up to two points, with the maximum possible score of nine [72]. Of the 17 observational study publications assessed (see Supplementary Materials Table S6), 2 were judged to be of high quality due to their representativeness of the average patient population and the assessment of the outcomes (e.g., all or >95% of subjects were accounted for at all follow-up points) [45,68]. Ten studies were assessed to be of fair quality [42,44,46,56,57,58,59,65,66,67]. The remaining six studies were judged to have an elevated risk of bias due to the outcome of interest not being reported at baseline or prior to intervention and/or due to insufficient reporting of the follow-up period [39,47,55,60,61,64].

## 3. Discussion

This SLR identified 31 unique studies from 33 publications that assessed the use of onabotA in the management of pain in 2740 patients with spasticity of various etiologies. Twenty-seven studies showed a reduction in pain following treatment with onabotA compared to baseline in adults with spasticity. OnabotA reduced pain consistently in patients with spasticity due to different etiologies, including stroke, MS, and CP, across all pain scales used and over multiple time points. The included studies had a high degree of heterogeneity in terms of study design, pain scale used, measure of pain (e.g., frequency, severity, and interference), patient population, dose of onabotA, baseline assessments, and time of follow-up. Due to this heterogeneity, it was not possible to conduct a meta-analysis on the effect of onabotA on pain related to spasticity. Despite this, there was consistency in the findings reported. Statistically significant improvement in pain following treatment with onabotA compared to baseline was reported in 12 studies in patients with spasticity of varying etiologies.

Although statistical significance is important in assessing the benefits of onabotA for the management of spasticity-related pain, it is also relevant to consider clinically meaningful changes for patients. In the literature, a clinically meaningful reduction in pain is defined as a 30% reduction from baseline [71]. A review of the studies reporting mean and median change in pain from baseline found 16 studies that met the criteria for clinically meaningful reduction in pain with onabotA compared to baseline. Four weeks was the most common follow-up time when a clinically meaningful reduction in pain was demonstrated, and this was reported in seven studies [14,51,54,58,65,66,67]. In an injection cycle, there is a typical peak effect at weeks 4–6 and a trough effect at roughly 12 weeks when, typically, repeat treatment may occur. Although it is difficult to draw definite conclusions from this analysis due to heterogeneity, these findings support the previous literature reporting a peak effect of onabotA at 4 weeks post-treatment [73,74].

Due to the heterogenous patient populations in the included studies, it was difficult to assess the impact of onabotA on pain in relation to the location of pain or etiology of spasticity. Location of pain was only specified in 6 of 31 studies, with 3 reporting shoulder pain, 1 reporting wrist pain, and 2 studies reporting pain from a range of different muscle groups [14,41,52,56,58,59]. Furthermore, the majority of studies in this SLR focused on post-stroke patients with spasticity while studies including other patient populations were limited. Thus, it was not possible to draw conclusions about differences in spasticity-related pain across these conditions.

Currently, there are three major BoNT/A formulations (onabotA, abobotulinumtoxinA, and incobotulinumtoxinA) [31,32,33] approved across a number of indications [34,35,36]. These formulations have unique physiochemical characteristics that result in distinct interactions with the tissue microenvironments into which they are injected [37]. Each formulation’s characteristics are due to differences in various steps of the manufacturing process (bacterial strain, fermentation, purification, excipients, finishing, and unit potency testing), all of which affect the clinical profile [37]. Units of BoNT/A products are not interchangeable due to differences in manufacturing and the assays used to measure unit potency, including different potency reference standards. Each BoNT/A has its own dosing information based on clinical studies in each indication; there are no established fixed inter-product dose ratios. In addition, study differences contribute to the variability among products. Study outcomes, including efficacy and duration of treatment, depend on specific assessments and definitions of response [37]. Moreover, all BoNT/A products exhibit product-specific dose responses that must be considered when comparing clinical properties such as duration. These study-level differences compound the intrinsic product-level differences, leading to unique clinical characteristics for each BoNT/A; thus, the results of this SLR in relation to onabotA cannot be extrapolated to other toxins [37].

A strength of this SLR is the large sample size of 2740 patients who were treated with onabotA. However, the reliability and validity of results may be impacted by the sample size of each study. Sample sizes in this SLR ranged from 5 to 530 patients [44,58] and, although no correlation between sample size and the significance of results was identified, small samples sizes can distort findings due to inadequate power and biased sampling [75]. Large patient populations were reported in the ASPIRE study (n = 484 and n = 530 upper limb and lower limb spasticity patients, respectively) [44,45], the InTENSE study (n = 140) [49,50], and a double-blind, multicenter study examining the efficacy of onabotA compared to Coretox (n = 220 patients) [51]. All three studies showed onabotA reduced pain compared to baseline, with two studies showing statistically significant reductions [44,45,51]. The statistical significance reported in two of the three largest studies supports the overall positive impact of onabotA on pain in patients with spasticity.

It is also important to assess if the study populations are representative of the broader population of patients with spasticity-related pain. The ASPIRE study includes patients with a range of etiologies, including stroke, MS, SCI, and TBI, regardless of previous onabotA treatment [44,45], and is considered to be representative of the broader patient population. Despite having a large patient population, other studies such as Lee et al. restricted participants with strict inclusion and exclusion criteria [51] and thus may not reflect the broader patient population. Although this SLR did not restrict patients with spasticity of different etiologies, the majority of studies assessed patients with post-stroke spasticity, and studies including other populations were limited. The evidence shows onabotA reduced pain in patients with spasticity across a range of etiologies; however, caution must be taken when extrapolating the findings to the broader population of spasticity patients. Previous studies have shown that post-stroke patients more frequently experience spasticity in the upper limbs compared to lower limbs and often with greater severity [76,77,78]. To our knowledge, there is no evidence assessing whether spasticity-associated pain differs depending on spasticity etiology, site (upper or lower limb), or type of pain (neuropathic, nociceptive, or mixed).

For effective pain management, valid and reliable assessment of pain including different types of pain is essential. The nature of pain makes objective measurement difficult; therefore, the use of one-dimensional tools such as VAS, NRS, and DAS is a popular method for reliable assessment of pain [79,80,81,82,83,84,85]. VAS provides a fast and convenient method of measuring pain and has been identified as a reliable tool that is sensitive to minor changes using a 0–100 scale [82]. The NRS scale, like VAS, has good reliability in the assessment of pain [82]. The sensitivity of NRS is dependent on the scale range. The majority of studies in this SLR used a 0–10 score range, which has been shown to be more sensitive to minor changes in pain compared to a 0–4 scale. Unlike VAS and NRS, the DAS is completed by clinical investigators rather than patients, with data obtained through patient interview [80]. DAS is therefore suitable for patients with impairment or functional disabilities. The DAS also has good reliability [79]. A study examining 10 experienced medical professionals assessing functional disability in post-stroke upper limb spasticity patients showed excellent DAS intra-rater reliability (classified as k ≥ 0.75) of assessments across two evaluations (Kendall W = 0.772, 95% CI 0.366–1.00, *p* < 0.001) and excellent inter-rater reliability between all 10 medical professionals (k = 0.776, 95% CI 0.533–1.00) [79].

This literature search was conducted up to June 2023; however, it is acknowledged that research evolves. Three studies published since completion of the SLR have been identified that would meet the inclusion criteria. Two of these studies support the findings here that onabotA improves pain in adults with spasticity [86,87], and one paper reported no improvement in any measured outcome, including pain, in patients with chronic stroke and severe activity limitations [88].

This SLR had a number of limitations which should be considered when reviewing the findings. Pain was not a primary outcome in many of the published studies and, therefore, statistical analysis was not always performed and was often reported inconsistently. In addition, studies did not evaluate what types of pain were treated with onabotA, e.g., neuropathic, nociceptive, or mixed. Previous studies have shown that post-stroke patients more regularly experience spasticity in the upper extremities compared to lower limbs and sometimes with greater severity [76,77,78]. To the best of our knowledge, there is no evidence examining if spasticity-associated pain differs depending on spasticity etiology or location of pain. Pain is a subjective experience, which makes objective measurement difficult; therefore, the use of one-dimensional tools is a popular method for reliable pain assessment.

In this review, a range of different pain scales were used to measure pain across studies; each pain scale has its own scoring system, range, dimensions (e.g., intensity, frequency, and interference with daily activities), and interpretation, which limits the comparability and reliability of pain outcomes and creates challenges when analyzing the results across studies. In addition, the pain scales may not be equally sensitive or specific in capturing the effects of onabotA on pain relief. Individual pain results need to be interpreted in the context of each scale’s responsiveness, validity, and reliability. Furthermore, instruments that include a single pain item (e.g., DAS, EQ-5D, and SF-36) have not been extensively studied outside of the functional disability score and there is limited evidence on the validity and reliability of pain assessment as a separate entity. Therefore, caution must be taken when interpreting isolated pain scores. Furthermore, DAS lacks the sensitivity of other pain assessment scales such as VAS and NRS, as it utilizes a narrow range of 0–3, which may not capture minor changes in patients’ pain. The DAS was also developed specifically for assessing disability in upper limb spasticity and, therefore, measurements applied to lower limb spasticity should be interpreted with caution. Variation in the application, implementation, and recall timeframe of the NRS in the studies reviewed make it difficult to collectively assess the reliability of pain assessment. A limitation of the NRS is the different interpretation of anchoring terms between patients [80,81]; however, it is assumed interpretation is consistent for individual patients across visits. Limitations of the VAS include that the scale is not self-explanatory, and, similar to the NRS, anchoring terms can be interpreted differently according to the age or cultural background of the respondent [80,81]. However, there are limited instruments that specifically assess pain related to spasticity currently; thus, there is a need for a standardized approach to measuring the effect of treatments on pain associated with spasticity.

A further limitation is that the included studies contained patients with various underlying conditions leading to spasticity; while this diversity reflects real-world clinical practice, it can also introduce heterogeneity in patient characteristics, potentially affecting the consistency of outcomes. The majority of the studies included patients with post-stroke spasticity, as studies including patients with spasticity due to other etiologies were limited. Additionally, many of the included studies were observational or open-label with limited blinding and small sample sizes. Observational studies, lack of blinding, and incomplete reporting of loss to follow-up may introduce confounding factors that affect the reliability of the findings. The heterogeneity in study design and blinding also make it challenging to directly compare and combine their results. Finally, although there may be some tendency for publication bias towards positive findings, this is less likely as pain was not the primary outcome in the studies reviewed.

## 4. Conclusions

The published evidence in this SLR shows an overall positive trend for reducing pain following the use of onabotA in adults with spasticity. Notwithstanding the limitations of the data across different etiologies, anatomical sites, rating scales, and trial designs, several conclusions are clear:

- The key findings highlight consistent positive effects of onabotA on pain reduction across different pain scales and diverse patient populations with spasticity, e.g., stroke, MS, and CP.
- OnabotA intramuscular injections are an effective, well-tolerated treatment in the management of spasticity-related pain.
- Time points of assessment varied considerably across studies (1 to 96 weeks, with 12 weeks most commonly assessed), but this had no observable impact on the effect of onabotA on pain.
- Overall, 17 publications demonstrated a clinically meaningful (≥30%) reduction in pain with onabotA compared to baseline at some or all time points, ranging from 1 to 84 weeks.
- Studies were heterogeneous and used a range of subjective measures of pain. There is a need for future research to identify a standardized measure of spasticity-associated pain.

## 5. Materials and Methods

### 5.1. Search Methods

The SLR was conducted and reported in accordance with the Preferred Reporting Items for Systematic Reviews and Meta-Analyses (PRISMA) guidelines. Databases searched included MEDLINE^®^ (via PubMed.com), Web of Science™ (via Clarivate™), and Cochrane Database of Systematic Reviews and Cochrane Controlled Register of Trials (via Cochrane Library). The searches were conducted on 20 June 2023 (for the full search strings, see Supplementary Materials Tables S1–S3). The bibliographies of the included studies were reviewed to obtain further relevant references. Additionally, ClinicalTrials.gov was reviewed to ensure that publications from ongoing or completed studies were captured. Six of the most visible international conference proceedings were searched, including the International Society of Physical and Rehabilitation Medicine (ISPRM), World Congress of Neurorehabilitation (WCNR), American Academy of Physical Medicine and Rehab (AAPM&R), American Academy of Neurology (AAN), International Movement Disorders Society—Parkinsonism and Related Disorders (MDS), and TOXINS (International Neurotoxin Association). Articles published between 1990 and June 2023 and conference presentations published between 2020 and 2023 were included. Results were limited to English language and the SLR protocol was registered on PROSPERO (reference CRD42023444039). There were no protocol amendments or deviations following PROSPERO registration.

### 5.2. Study Selection

Papers for inclusion were identified by evaluating the retrieved publications against the pre-determined PICOS-T criteria (Table 1).

Titles and abstracts identified from the database searches were screened by two independent reviewers to assess suitability for inclusion in the SLR, according to the pre-defined inclusion and exclusion criteria. Discrepancies were resolved by a third reviewer who independently reviewed the title and abstract for inclusion. Full-text screening of the identified papers was then performed by two independent reviewers and discrepancies independently resolved by a third reviewer. Multiple reports of the same study were linked before data extraction using available information, e.g., trial registration numbers, authors’ names, study sponsors, location, participants, etc. After linking publications, reviewers determined the primary publication for extracting data for each study.

Data from the eligible papers was extracted by a single reviewer and independently validated by a second reviewer into a data extraction template (DET) designed in Microsoft Excel™. A third reviewer was consulted to resolve disagreements where necessary. The quality of included studies was assessed using standard quality appraisal checklists: Cochrane Risk-of-Bias Tool v2.0 for RCTs [89] and Newcastle–Ottawa Scale for non-randomized studies [72]. Structured summaries were used to synthesize and summarize the data, as alternative synthesis methods such as meta-analysis were not possible due to study heterogeneity.

## Figures and Tables

**Table 1:** PICOS-T selection criteria for identification of relevant studies.

Criteria	Inclusion Criteria	Exclusion Criteria
Population	Adults (≥18 years) with (any) spasticity (upper and/or lower limb)	Patients <18 years old Pregnant or breastfeeding women Animals/in vitro studies
Intervention	Studies involving onabotulinumtoxinA (BOTOX^®^, onabotulinum, onabotA, OBT-A)	Studies not evaluating onabotA
Comparator	Any comparator	None
Outcomes	Studies reporting on the following clinical outcomes for the population of interest were included: Pain (severity and frequency) * McGill pain questionnaire Visual analog scale Brief pain inventory Pain measured with DAS or EQ-5D scale NRS to assess pain Disability and HRQoL scales to assess pain	Studies not reporting at least one of the outcomes of interest
Study design/publication type	Randomized and non-randomized controlled trials Observational studies (prospective and retrospective cross-sectional studies, cohort studies, case–control studies, patient surveys, patient registries, patient records, medical chart reviews)	Preclinical studies Reviews, letters, comments, and editorials Case reports Studies not in English
Timeframe	Full texts published from 1990 to June 2023 Congress/meeting abstracts published from 2020 to 2023	Full texts published before 1990 Abstracts published before 2020

* Only studies examining the effect of onabotA on pain outcomes in patients with spasticity were included; pain in relation to adverse events/injection sites were not recorded. DAS, Disability Assessment Scale; EQ-5D, EuroQoL 5-Dimension; HRQoL, health-related quality of life; NRS, numerical rating scale; PICOS-T, Population, Intervention, Comparator, Outcomes, Study Design-Timeframe.

**Table 2:** Characteristics of the studies included in the systematic literature review.

Author, Year	Study Design	Population	Cohort	Country	Pain Scale Description
Bergfeldt 2006 [39]	Observational (retrospective analysis)	Focal spasticity in patients with CP, stroke, and TBI	Overall cohort (n = 100)	Sweden	VAS (details NR)
Childers 2004 [40]	RCT (double-blind, placebo-controlled)	Post-stroke focal upper limb spasticity	Placebo (n = 26) OnabotA 90U (n = 21) OnabotA 180U (n = 23) OnabotA 360U (n = 21)	United States	5-point frequency of pain scale (0 = never, 4 = constant) 5-point severity of pain scale (0 = none, 4 = very severe/intolerable)
deBoer 2008 [41]	RCT (double-blind, placebo-controlled)	Stroke patients with spastic hemiplegia	OnabotA (n = 10) Placebo (n = 11)	The Netherlands	VAS (0–10 cm, 0 = no pain, 10 = worst pain)
De Icco 2019 [42]	Observational (open-label study)	Stroke, MS, and SCI with spasticity and pain with neuropathic features	Overall cohort (n = 25) Stroke (n = 10) MS (n = 10) SCI (n = 5)	Italy	NPSI NRS (0–10, 0 = no sensation, 10 = unbearable pain, with pain threshold verbally anchored to 5)
Devier 2017 [43]	RCT (single-blind)	Post-stroke upper limb spasticity	OnabotA + Rehab (n = 15) OnabotA (n = 16)	United States	Fugl-Meyer subscale rating pain during passive ROM
ASPIRE, Esquenazi 2021 * [44]	Observational (multicenter, prospective, observational registry)	Adults across multiple etiologies with lower limb spasticity related to upper motor neuron syndrome	Overall cohort (n = 530)	France, Germany, Italy, Spain, Taiwan, United States, United Kingdom	DAS (0–3, 0 = no disability, 3 = severe disability) NRS (0–10, 0 = no sensation, 10 = unbearable pain)
ASPIRE, Francisco 2020 * [45]	Observational (multicenter, prospective, observational registry)	Adults across multiple etiologies with upper limb spasticity related to upper motor neuron syndrome	Overall cohort (n = 484)	France, Germany, Italy, Spain, Taiwan, United States, United Kingdom	DAS (0–3, 0 = no disability, 3 = severe disability) NRS (0–10, 0 = no sensation, 10 = unbearable pain)
Gordon 2004 [46]	Observational (open label)	Post-stroke spasticity	Overall cohort (n = 111)	United States	DAS (0–3, 0 = no disability, 3 = severe disability)
Jog 2016 [47]	Observational (prospective)	Adult focal spasticity, blepharospasm, cerebral palsy, cervical dystonia, hemifacial spasm, and hyperhidrosis	Adult focal spasticity: OnabotA naïve (n = 151) OnabotA maintenance (n = 247) CP: OnabotA naïve (n = 4) OnabotA maintenance (n = 18)	Canada	SF-6D (bodily pain sub-domain)
Kaji 2010 [48]	RCT (double-blind, parallel-group, placebo-controlled)	Post-stroke upper limb spasticity	High-dose onabotA (n = 51) High-dose placebo (n = 26) Low-dose onabotA (n = 21) Low-dose placebo (n = 11)	Japan	DAS (0–3, 0 = no disability, 3 = severe disability)
InTENSE, Lannin 2020 ** [49] Lannin 2022 ** [50]	RCT (phase III, single-blind)	Post-stroke patients with upper limb spasticity	Overall cohort (n = 140)	Australia	VAS (0–10 cm, 0 = no pain, 10 = worst pain)
Lee 2020 [51]	RCT (double-blind, active drug-controlled, phase III clinical)	Post-stroke upper limb spasticity	OnabotA (n = 109) Coretox^®^ (n = 110)	Republic of Korea	DAS (0–3, 0 = no disability, 3 = severe disability)
Lim 2008 [52]	RCT (double-blind, comparative)	Patients with hemiplegic shoulder pain	OnabotA (n = 16) Triamcinolone acetonide (n = 13)	South Korea	NRS (on a scale of 0–10, where 0 = no pain and 10 = highest pain level) during passive ROM of the shoulder in four planes (forward flexion, abduction, external and internal rotation)
Maanum 2011 [53]	RCT (double-blind, placebo-controlled)	Adults with spastic cerebral palsy	OnabotA (n = 33) Placebo (n = 33)	Norway	SF-36 (bodily pain sub-domain)
Mancini 2005 [54]	RCT (double-blind, dose-ranging)	Lower limb post-stroke spasticity	Low-dose onabotA (n = 15) Medium-dose onabotA (n = 15) High-dose onabotA (n = 15)	Italy	VAS (0–10 cm, 0 = no pain, 10 = worst pain)
Marciniak 2008 [55]	Observational (retrospective chart review)	SCI receiving their first injection of onabotA for spasticity control	Overall cohort (n = 28)	United States	Patient-reported improvement
Marciniak 2012 [14]	RCT (double-blind, placebo-controlled)	Post-stroke patients reporting pain associated with tightness of the shoulder muscles	OnabotA (n = 10) Control (saline) (n = 11)	United States	DAS (0–3, 0 = no disability, 3 = severe disability) MPQ (0–78, 0 = no pain, 78 = excruciating pain) VAS (0–10 cm, 0 = no pain, 10 = worst pain; assessing pain at its best, worst, pain with upper body dressing, and sleep interference caused by pain)
Miscio 2004 [56]	Observational	Chronic post-stroke patients with wrist spasticity	OnabotA (n = 12) AbobotulinumtoxinA (Dysport^®^) (n = 6)	Italy	VAS (0–10 cm, 0 = no pain, 10 = worst pain)
Reiter 1996 [57]	Observational (open-label, single-arm, single-blind)	Patients with post-stroke spasticity	Overall cohort (n = 17)	Italy	VAS (details NR)
Restivo 2003 [58]	Observational	Patients with MS and painful tonic spasms	Overall cohort (n = 5)	Italy	4-point intensity of pain score (0 = no pain, 1 = mild, 2 = moderate, 3 = severe)
Rousseaux 2002 [59]	Observational	Hemiplegic patients resulting from stroke	Overall cohort (n = 20)	France	NRS (0–6, 0 = no pain, 6 = permanent and unbearable pain)
Rousseaux 2014 [60]	Observational (open-label)	Patients who had suffered a unilateral stroke or TBI with disabling lower limb flexion	Overall cohort (n = 11)	France	VAS (0–10, 0 = no pain, 10 = unbearable pain)
Sampaio 1997 [61]	Observational (phase III, open-label)	Patients with arm spasticity due to stroke	Overall cohort (n = 19)	Portugal	NRS of pain severity (0 = best score, 5 = worst score)
Seo 2015 [62]	RCT (double-blind, active drug-controlled, phase III)	Stroke patients with moderate to severe upper limb spasticity	OnabotA (n = 98) Neuronox^®^ (n = 94)	South Korea	DAS (0–3, 0 = no disability, 3 = severe disability)
Simpson 1996 [63]	RCT (graduated dose, double-blind, parallel-group, placebo-controlled)	Patients at least 9 months post-stroke with upper limb spasticity	Low-dose onabotA (75U) (n = 9) Medium-dose onabotA (150U) (n = 9) High-dose onabotA (300U) (n = 9) Placebo (n = 10)	United States	Pain assessment †
Slawek 2005 [64]	Observational (open-label, prospective)	Stroke patients with upper limb spasticity	Overall cohort (n = 21)	Poland	VAS (details NR)
Suputtitada 2002 [65]	Observational (open-label, prospective)	Hemiplegic patients with spastic toes	Overall cohort (n = 20)	Thailand	VAS (0–100, 0 = no pain and 100 = maximum pain)
Turhanoglu 2002 [66]	Observational (open-label)	Patients with spasticity from stroke-related hemiplegia, transverse myelitis, and MS	Overall cohort (n = 23)	Turkey	VAS (0–10, 0 = no pain and 10 = worst pain imaginable)
Wang 2002 [67]	Observational (open-label, non-controlled)	Patients with post-stroke upper limb spasticity and dysfunction undergoing rehabilitation	Overall cohort (n = 16)	China	Limb pain (0–10, 0 = no pain and 10 = maximum pain)
Wissel 2000 [68]	Observational (prospective)	Patients with upper and/or lower limb spasticity due to upper motor neuron syndrome and pain as the primary spasticity-related complaint	Overall cohort (n = 60) Acute spasticity (n = 17) Chronic spasticity (n = 43)	Germany, Austria	Patient Evaluation of Global Response to OnabotA Treatment: -4 = Marked worsening in severity of pain and functioning -3 = Moderate worsening severity of pain causing decline in function -2 = Moderate worsening severity of pain, no change in function -1 = Mild worsening in severity of pain, no change in function 0 = No effect +1 = Mild improvement in severity of pain, no change in function +2 = Moderate improvement in severity of pain, no change in function +3 = Moderate improvement in severity of pain causing functional improvement +4 = Marked improvement in severity of pain and in function
Wissel 2016 [69]	RCT (double-blind, placebo-controlled)	Patients with post-stroke spasticity	OnabotA + standard care (n = 139) Placebo + standard care (n = 134)	Germany, Sweden, United Kingdom, Canada	NRS (0–10, 0 = no pain and 10 = worst imaginable pain)
Zeuner 2017 [70]	RCT (single-blind)	Patients with post-stroke spasticity	Ultrasound-guided injection (n = 5) Electromyographic-guided injection (n = 7) Control (n = 11)	Germany	DAS (0–3, 0 = no disability, 3 = severe disability), EQ-5D

* Esquenazi 2021 and Francisco 2020 present results from the ASPIRE study but from different populations, i.e., upper or lower limb. ** Lannin 2020 and Lannin 2022 present results from the same study but with different follow-up times, i.e., 3 months or 12 months. † No additional details of pain assessment conducted in the study are reported in Simpson et al. 1996. ASPIRE, Adult SPasticity International Registry; DAS, disability assessment scale; cm, centimeter; CP, cerebral palsy; EQ-5D, EuroQol instrument; InTENSE, Intensive Therapy Efficacy After Neurological Spasticity Treatment; MPQ, McGill pain questionnaire; MS, multiple sclerosis; NPSI, neuropathic pain symptom inventory; NR, not reported; NRS, numerical rating scale; OnabotA, onabotulinumtoxinA; RCT, randomized controlled trial; ROM, range of motion; SF-36, short-form 36-items survey; SCI, spinal cord injury; SF-6D, short-form six-dimension; TBI, traumatic brain injury; VAS, visual analog scale.

**Table 3:** Cochrane Risk of Bias (v2.0) assessment for the included RCTs.

Author, Year	Randomization Process	Deviations from Intended Interventions	Missing Outcome Data	Measurement of the Outcome	Selection of the Reported Results	Overall Bias
Childers 2004 [40]	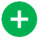	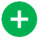	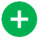	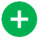	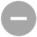	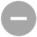
De Boer 2008 [41]	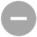	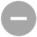	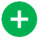	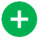	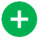	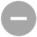
Devier 2017 [43]	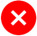	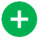	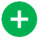	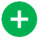	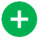	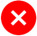
Kaji 2020 [48]	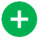	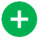	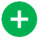	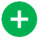	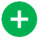	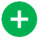
Lannin 2020 [49]	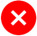	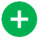	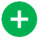	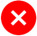	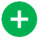	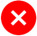
Lannin 2022 [50]	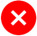	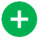	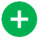	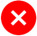	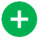	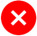
Lee 2020 [51]	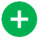	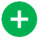	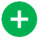	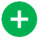	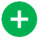	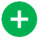
Lim 2008 [52]	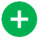	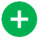	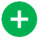	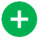	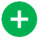	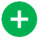
Maanum 2011 [53]	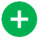	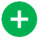	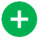	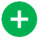	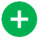	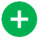
Mancini 2005 [54]	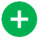	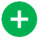	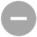	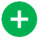	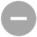	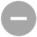
Marciniak 2012 [14]	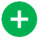	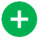	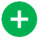	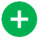	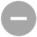	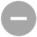
Seo 2015 [62]	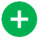	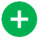	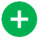	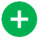	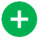	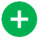
Simpson 1996 [63]	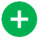	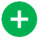	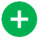	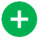	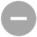	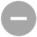
Wissel 2016 [69]	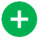	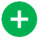	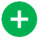	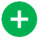	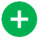	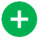
Zeuner 2017 [70]	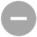	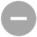	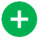	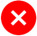	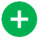	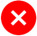
High risk 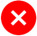		Some concerns 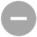		Low risk 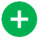		

RCT, randomized controlled trials.

## Data Availability

As this was a systematic literature review, no new data were created or analyzed in this study. Data sharing is not applicable to this article.

## Supplementary Materials

The following supporting information can be downloaded at: https://www.mdpi.com/article/10.3390/toxins17080418/s1, Table S1: Medline search strategy via PubMed; Table S2: Embase search strategy via Embase; Table S3: Cochrane Library search strategy via Cochrane Library; Table S4: Pain outcomes using the Visual Analog Scale; Table S5: Pain outcomes using the Numerical Rating Scale; Table S6: Newcastle-Ottawa Scale–Assessment of included observational studies.
