# Supplementary material for: OnabotulinumtoxinA in the Management of Pain in Adult Patients with Spasticity: A Systematic Literature Review
Source: Toxins (Basel). 2025 Aug 18;17(8):418. doi: 10.3390/toxins17080418 (PMC12390114; doi:10.3390/toxins17080418)
Supplement: Supplementary file 1 [file toxins-17-00418-s001.zip › toxins-3754626-supplementary.pdf]

Supplementary Materials: OnabotulinumtoxinA in the Management of Pain in Adult Patients with Spasticity: A Systematic Literature Review

Francesca Bianchi, Mariana Nelson, Jörg Wissel, Heakyung Kim, Alexandra Traut, Darshini Shah, Tiziana Musacchio and Bahman Jabbari

Table S1. Medline search strategy via PubMed

No.	Keywords	Search terms	Results
**#1**	Population	“muscle spasticity" [MeSH Terms] OR ("muscle" [All Fields] AND "spasticity"[All Fields]) OR "muscle spasticity"[All Fields] OR "spastic"[All Fields] OR "spasticity"[All Fields] OR "spastics"[All Fields] OR "spasticities"[All Fields]	32,840
**#2**		"muscle contraction"[MeSH Terms] OR ("muscle"[All Fields] AND "contraction"[All Fields]) OR "muscle contraction"[All Fields] OR "contractions"[All Fields]	260,584
**#3**		"spasm"[MeSH Terms] OR "spasm"[All Fields] OR ("muscle"[All Fields] AND "spasm"[All Fields]) OR "muscle spasm"[All Fields] OR "spasm"[MeSH Terms]	27,471
**#4**		"stiff"[All Fields] OR "stiffness"[All Fields] OR "stiffnesses"[All Fields]	96,668
**#5**		"dystonia"[MeSH Terms] OR "dystonia"[All Fields] OR "dystonias"[All Fields] OR "dystonic disorders"[MeSH Terms] OR ("dystonic"[All Fields] AND "disorders"[All Fields]) OR "dystonic disorders"[All Fields]	23,271
**#6**		"muscle rigidity"[MeSH Terms] OR ("muscle"[All Fields] AND "rigidity"[All Fields]) OR "muscle rigidity"[All Fields]	4,477
**#7**		#1 OR #2 OR #3 OR #4 OR #5 OR #6	429,836
**#8**		headache*[Title] OR migraine*[Title] OR "bladder"[Title] OR "blepharospasm*"[Title] OR "Hyperhidrosis"[Title] OR "cosmetic*"[Title] OR "urinary"[Title] OR "neuralgia*"[Title] OR "bruxism"[Title] OR "myofascial"[Title] OR "pelvic"[Title]	320,556
**#9**		#7 NOT #8	421,116
**#10**	Intervention	"botulinum toxins, type a"[MeSH Terms] OR "type a botulinum toxins"[All Fields] OR "botulinum"[All Fields] OR "bonta"[All Fields] OR "bont-a"[All Fields]	27,683
**#11**		"allergan"[All Fields] OR "abbvie"[All Fields]	39,335
**#12**		#10 AND #11	779
**#13**		"onabotulinum"[All Fields] OR "OnabotulinumtoxinA"[All Fields] OR "onabotA"[All Fields] OR "OBT-A"[All Fields] OR "botox"[All Fields] OR "vistabel"[All Fields]	3,810
**#14**		#12 OR #13	4,138
**#15**		#9 AND #14	823
**#16**	Study design	"clinical trial, veterinary"[Publication Type]	7,133
**#17**		"case reports"[Publication Type] OR "case report"[Title]	2,388,004
**#18**		"case serie*"[Title]	31,886
**#19**		#16 OR #17 OR #18	2,415,974

 https://doi.org/10.3390/toxins17080418

#20		#15 NOT #19	731
#21		"letter"[Publication Type] OR "correspondence as topic"[MeSH Terms] OR "letter"[Title]	1,269,434
#22		"comment"[Publication Type] OR "commentary"[Title]	1,026,749
#23		"editorial"[Publication Type] OR "editorial"[Title]	675,855
#24		#21 OR #22 OR #23	2,243,062
#25		#20 NOT #24	722
#26	Language	Limit to English language	669
#27	Humans	Limit to human studies	526
#28	Timeframe	Limit from 1990 to 2023	525

Search conducted 20th June 2023

Table S2. Embase search strategy via Embase

No.	Keywords	Search Terms	Results
#1	Population	ALL=("muscle spasticity" OR ("muscle" AND "spasticity") OR "muscle spasticity" OR "spastic" OR "spasticity" OR "spastics" OR "spasticities")	25,710
#2		ALL=("muscle contraction" OR ("muscle" AND "contraction") OR "muscle contraction" OR "contractions")	84,302
#3		ALL=("spasm" OR "spasm" OR ("muscle" AND "spasm") OR "muscle spasm" OR muscle spasm)	11,680
#4		ALL=("stiff" OR "stiffness" OR "stiffnesses")	273,600
#5		ALL=("dystonia" OR "dystonia" OR "dystonias" OR "dystonic disorders" OR ("dystonic" AND "disorders") OR "dystonic disorders")	20,434
#6		ALL=("muscle rigidity" OR ("muscle" AND "rigidity") OR "muscle rigidity")	1,825
#7		#1 OR #2 OR #3 OR #4 OR #5 OR #6	408,090
#8		TI=(headache* OR migraine* OR "bladder" OR "blepharospasm*" OR "Hyperhidrosis" OR "cosmetic*" OR "urinary" OR "neuralgia*" OR "bruxism" OR "myofascial" OR "pelvic")	217,875
#9		#7 NOT #8	403,847
#10	Intervention	ALL=("botulinum toxins, type a" OR "type a botulinum toxins" OR "botulinum" OR "bonta" OR "bont-a")	27,300
#11		ALL=("allergan" OR "abbvie")	39,400
#12		#10 AND #11	1,210
#13		ALL=("onabotulinum" OR "OnabotulinumtoxinA" OR "onabotA" OR "OBT-A" OR "botox" OR "vistabel")	5,461

**#14**		#12 OR #13	6,096
**#15**		#9 AND #14	1,272
**#16**	Study design	WC=(Veterinary Sciences) OR TS=("veterinary clinical trial")	439,947
**#17**		TI=("case report*" OR "case serie*")	280,161
**#18**		#16 OR #17	716,617
**#19**		#15 NOT #18	1,241
**#20**		DT=(Letter OR Editorial Material OR Book OR Book Chapter OR Book Review OR Discussion)	3,592,556
**#21**		#19 NOT #20	1,171
**#22**	Language	Limit to English language and removal of PubMed duplicated studies	825

Search conducted 20th June 2023

Table S3. Cochrane Library search strategy via Cochrane Library

**No.**	**Keywords**	**Search Terms**	**Results**
**#1**	Population	MeSH descriptor: [Muscle Spasticity] explode all trees	1,137
**#2**		("muscle" AND "spasticity") OR "muscle spasticity" OR "spastic" OR "spasticity" OR "spastics" OR "spasticities"	5,411
**#3**		MeSH descriptor: [Muscle Contraction] explode all trees	8,442
**#4**		("muscle" AND "contraction") OR "muscle contraction" OR "contractions"	13,593
**#5**		MeSH descriptor: [Spasm] explode all trees	699
**#6**		"spasm" OR ("muscle" AND "spasm") OR "muscle spasm"	4,218
**#7**		"stiff" OR "stiffness" OR "stiffnesses"	13,214
**#8**		MeSH descriptor: [Dystonia] explode all trees	411
**#9**		MeSH descriptor: [Dystonic Disorders] explode all trees	164
**#10**		"dystonia" OR "dystonias" OR ("dystonic" AND "disorders") OR "dystonic disorders"	1,535
**#11**		MeSH descriptor: [Muscle Rigidity] explode all trees	137
**#12**		("muscle" AND "rigidity") OR "muscle rigidity"	993
**#13**		#1 OR #2 OR #3 OR #4 OR #5 OR #6 OR #7 OR #8 OR #9 OR #10 OR #11 OR #12	40,585
**#14**		(headache* OR migraine* OR bladder OR blepharospasm* OR Hyperhidrosis OR cosmetic* OR	38,062

		urinary OR neuralgia* OR bruxism OR myofascial OR pelvic):ti	
**#15**		#13 NOT #14	39,103
**#16**	Intervention	MeSH descriptor: [Botulinum Toxins, Type A] explode all trees	2,069
**#17**		"type a botulinum toxins" OR "type a botulinum toxin" OR "botulinum" OR "bonta" OR "bont-a"	5,063
**#18**		"allergan" OR "abbvie"	2,573
**#19**		((#16 OR #17) AND (#18))	233
**#20**		"onabotulinum" OR "OnabotulinumtoxinA" OR "onabotA" OR "OBT-A" OR "botox" OR "vistabel"	2,133
**#21**		#19 AND #20	204
**#22**		#15 AND #21	62
**#23**	Humans	Limit to human studies	2,967
**#24**	Timeframe	Limit from 1990 to 2023, in trials	47
**#25**	Database	Limit from 1990 to 2023, in Cochrane Reviews	13

Search conducted 20th June 2023

Table S4. Pain outcomes using the Visual Analog Scale

First author, year	Cohort	Time-point of post-intervention measurement	Baseline mean (SD) / median (range) value	Post-intervention value, mean (SD)	Change from baseline, mean (SD)	P-value
Bergfeldt 2006*	Overall cohort	6 weeks	NR	NR	NR	NR
deBoer 2008	OnabotA	6 weeks	44.9 (15.2)	**42.5†**	NR	p=0.08 average improvement pre- vs post-intervention
		12 weeks		**38.1 (18.2)**	NR	
	Placebo	6 weeks	61.7 (23.2)	**48.1†**	NR	
		12 weeks		**46.8 (27.2)**	NR	
InTENSE study<br>• Lannin 2020<br>• Lannin 2022	Overall cohort	3 months	1.9 (2.6)	**1.0 (2.0)**	**-0.9**	NR
		12 months		**1.1 (2.0)**	**-0.8**	NR
	Experimental	3 months	1.9 (2.7)	**1.0 (2.0)**	**-0.9 (2.5)**	NR
		12 months		1.1 (2.1)	**-0.6 (2.5)**	NR
	Control	3 months	1.9 (2.6)	**1.1 (2.0)**	**-0.9 (2.6)**	NR
		12 months		**1.1 (1.9)**	**-0.9 (2.4)**	NR
	Received further onabotA	12 months	1.0 (1.8)	**0.7 (1.2)**	**-0.4 (2.1)**	NR
	Did not receive further onabotA	12 months	2.2 (2.8)	**1.2 (2.2)**	**-0.9 (2.6)**	NR
Mancini 2005	Low onabotA dosage	4 weeks	18.82†	**4.72†**	NR	NR
		4 months		**4.72†**	NR	NR
	Medium onabotA dosage	4 weeks	23.19†	**6.81†**	NR	NR
		4 months		**12.01†**	NR	NR
	High onabotA dosage	4 weeks	17.29†	**8.47†**	NR	NR
		4 months		**6.94†**	NR	NR
Marciniak 2012	OnabotA - Pain at best	2 weeks	24.83 (20.63 - 42.57)	NR	**-5.17 (-13.23 to 1.74)**	0.481 (onabotA vs placebo)
	Saline/control - Pain at best	2 weeks	19.94 (0.29 - 55.88)	NR	0.84 (-2.93 to 5.60)	0.481 (onabotA vs placebo)
	OnabotA - Pain at best	4 weeks	24.83 (20.63 - 42.57)	NR	**-6.01 (-19.09 to 5.57)**	0.684 (onabotA vs placebo)

First author, year	Cohort	Time-point of post-intervention measurement	Baseline mean (SD) / median (range) value	Post-intervention value, mean (SD)	Change from baseline, mean (SD)	P-value
	Saline/control - Pain at best	4 weeks	19.94 (0.29 - 55.88)	NR	1.46 (-3.50 to 5.34)	0.684 (onabotA vs placebo)
	OnabotA - Pain at best	12 weeks	24.83 (20.63 - 42.57)	NR	**-5.91 (-9.56 to 27.34)**	0.912 (onabotA vs placebo)
	Saline/control - Pain at best	12 weeks	19.94 (0.29 - 55.88)	NR	5.51 (-4.86 to 17.57)	0.912 (onabotA vs placebo)
	OnabotA - Pain at worst	2 weeks	53.00 (43.90 - 64.00)	NR	**-12.29 (-29.30 to -1.43)**	0.684 (onabotA vs placebo)
	Saline/control - Pain at worst	2 weeks	70.72 (27.80 - 81.88)	NR	**-17.16 (-3.90 to -5.71)**	0.684 (onabotA vs placebo)
	OnabotA - Pain at worst	4 weeks	53.00 (43.90 - 64.00)	NR	**-18.64 (-29.73 to 2.14)**	0.529 (onabotA vs placebo)
	Saline/control - Pain at worst	4 weeks	70.72 (27.80 - 81.88)	NR	**-19.88 (-69.90 to -2.16)**	0.529 (onabotA vs placebo)
	OnabotA - Pain at worst	12 weeks	53.00 (43.90 - 64.00)	NR	**-29.07 (-50.20 to -13.27)**	0.796 (onabotA vs placebo)
	Saline/control - Pain at worst	12 weeks	70.72 (27.80 - 81.88)	NR	**-29.98 (73.88 to -15.75)**	0.796 (onabotA vs placebo)
	OnabotA - Pain with dressing	2 weeks	29.10 (17.70 - 30.71)	NR	**-6.97 (-25.29 to 10.43)**	0.796 (onabotA vs placebo)
	Control/saline – Pain with dressing	2 weeks	46.50 (4.75 - 75.53)	NR	**-3.04 (-26.11 to -2.43)**	0.796 (onabotA vs placebo)
	OnabotA - Pain with dressing	4 weeks	29.10 (17.70 - 30.71)	NR	**-4.39 (-24.71 to 5.19)**	0.436 (onabotA vs placebo)
	Control/saline – Pain with dressing	4 weeks	46.50 (4.75 - 75.53)	NR	**-5.29 (-28.54 to 0.07)**	0.436 (onabotA vs placebo)
	OnabotA - Pain with dressing	12 weeks	29.10 (17.70 - 30.71)	NR	**-11.10 (-17.57 to 2.01)**	0.853 (onabotA vs placebo)

https://doi.org/10.3390/toxins17080418

First author, year	Cohort	Time-point of post-intervention measurement	Baseline mean (SD) / median (range) value	Post-intervention value, mean (SD)	Change from baseline, mean (SD)	P-value
	Control/saline – Pain with dressing	12 weeks	46.50 (4.75 - 75.53)	NR	**-5.32 (-25.25 to 0)**	0.853 (onabotA vs placebo)
	OnabotA - Pain interfering with sleep	2 weeks	33.84 (20.83 - 54.13)	NR	**-5.84 (-24.11 to 2.87)**	0.579 (onabotA vs placebo)
	Control/saline – Pain interfering with sleep	2 weeks	42.51 (5.88 - 58.92)	NR	**-2.11 (-20.71 to 13.16)**	0.579 (onabotA vs placebo)
	OnabotA - Pain interfering with sleep	4 weeks	33.84 (20.83 - 54.13)	NR	2.73 (-20.64 to 15.86)	0.247 (onabotA vs placebo)
	Control/saline – Pain interfering with sleep	4 weeks	42.51 (5.88 - 58.92)	NR	**-7.44 (-24.59 to 1.87)**	0.247 (onabotA vs placebo)
	OnabotA - Pain interfering with sleep	12 weeks	33.84 (20.83 - 54.13)	NR	**-3.09 (-17.89 to 6.47)**	0.739 (onabotA vs placebo)
	Control/saline – Pain interfering with sleep	12 weeks	42.51 (5.88 - 58.92)	NR	**-1.51 (-26.16 to 1.00)**	0.739 (onabotA vs placebo)
Miscio 2004	OnabotA	NR	8	**3**	NR	NR
	Dysport	NR	5.5	**0**	NR	NR
Reiter 1996	Overall cohort	1 month	NR	NR	NR	NR
Rousseaux 2014	Overall cohort	Week 10 ± 2 (session 3)	Session 1: Spontaneous pain: 1.1 (1.9); Mobilisation pain: 7.0 (2.8)	**Spontaneous pain: 0.3 (0.9) Mobilisation pain: 4.6 (2.1)**	NR	NR

First author, year	Cohort	Time-point of post-intervention measurement	Baseline mean (SD) / median (range) value	Post-intervention value, mean (SD)	Change from baseline, mean (SD)	P-value
			Session 2: Spontaneous pain: 1.3 (2.2); Mobilisation pain: 7.3 (2.8)			
		Week 21 ± 3 (session 4)		**Spontaneous pain: 0.5 (1.6) mobilisation pain: 4.8 (2.5)**	NR	Spontaneous pain: p=0.067 Mobilisation pain: p=0.0001 (Session 2 vs. session 3 or 4)
Slawek 2005	Overall cohort	NR	NR	NR	NR	NR
Suputtitada 2002	Overall cohort	4 weeks	82†	**17.2†**	NR	NR
		8 weeks		**0†**	NR	NR
		12 weeks		**3.4†**	NR	NR
		16 weeks		**6.2†**	NR	NR
		20 weeks		**25.4†**	NR	NR
		24 weeks		**34.2†**	NR	NR
Turhanoglu 2002	Overall cohort	1 week	7.84 (4.33)	**4.12 (2.84)**	NR	p<0.001
		1 month		**3.93 (1.68)**	NR	p<0.001
		3 months		**3.43 (1.78)**	NR	p<0.001

*Study reported patients with pain reduction/improvement, n (%): 30/32 (94); 95% CI: 79-99; †Estimate from figure. All post-intervention values that are lower than baseline are **highlighted in bold.**
NR, not reported; SD, standard deviation

Table S5. Pain outcomes using the Numerical Rating Scale

First author, year	Treatment cohort	Time-point of post-intervention measurement	Baseline mean value (SD)	Post-intervention value, mean (SD)	Change from baseline, mean (SD)	P-value
Childers 2004	Placebo	NR	Frequency score: 1.0<br>Intensity score: NR	NR	NR	NR
	Low dose onabotA 90 U) cohort	NR	Frequency score: 1.0<br>Intensity score: 1.3	NR	NR	NR
	Middle dose onabotA (180 U)	NR	Frequency score: 1.3<br>Intensity score: 1.5	NR	NR	NR
	High dose onabotA (360 U)	NR	Frequency score: 1.3<br>Intensity score: 1.5	NR	NR	NR
De Icco 2019	Overall cohort	30 days	2.5 (3.4)	**2.0 (2.9)**	NR	p=0.024 (pre- vs post-treatment)
Esquenazi 2021	OnabotA	Treatment session 1 (approx. 12 weeks)	4.0 (3.2)	NR	**−0.7 (3.0)**	0.0053 (pre- vs post-treatment)
		Treatment session 2 (approx. 24 weeks)	4.0 (3.2)	NR	**−1.2 (3.1)**	<0.0001 (pre- vs post-treatment)
		Treatment session 3 (approx. 36 weeks)	4.0 (3.2)	NR	**−1.4 (3.2)**	<0.0001 (pre- vs post-treatment)
		Treatment session 4 (approx. 48 weeks)	4.0 (3.2)	NR	**−0.8 (3.2)**	0.0022 (pre- vs post-treatment)

 https://doi.org/10.3390/toxins17080418

**First author, year**	**Treatment cohort**	**Time-point of post-intervention measurement**	**Baseline mean value (SD)**	**Post-intervention value, mean (SD)**	**Change from baseline, mean (SD)**	**P-value**
		Treatment session 5 (approx. 60 weeks)	4.0 (3.2)	NR	**−1.1 (3.2)**	<0.0001 (pre- vs post-treatment)
		Treatment session 6 (approx. 72 weeks)	4.0 (3.2)	NR	**−1.0 (3.0)**	0.0006 (pre- vs post-treatment)
		Treatment session 7 (approx. 84 weeks)	4.0 (3.2)	NR	**−1.3 (3.5)**	0.0019 (pre- vs post-treatment)
		Treatment session 8 (approx. 96 weeks)	4.0 (3.2)	NR	**−0.1 (2.9)**	0.8930 (pre- vs post-treatment)
Francisco 2020	OnabotA	Treatment session 1 (approx. 12 weeks)	3.8 (3.3)	NR	**−0.6**	NR
		Treatment session 2 (approx. 24 weeks)	3.8 (3.3)	NR	**−1.2**	<0.006 (pre- vs post-treatment)
		Treatment session 3 (approx. 36 weeks)	3.8 (3.3)	NR	**−1.1**	<0.006 (pre- vs post-treatment)
		Treatment session 4 (approx. 48 weeks)	3.8 (3.3)	NR	**−0.6**	NR
		Treatment session 5 (approx. 60 weeks)	3.8 (3.3)	NR	**−0.7**	NR
		Treatment session 6 (approx. 72 weeks)	3.8 (3.3)	NR	**−0.6**	NR

 https://doi.org/10.3390/toxins17080418

**First author, year**	**Treatment cohort**	**Time-point of post-intervention measurement**	**Baseline mean value (SD)**	**Post-intervention value, mean (SD)**	**Change from baseline, mean (SD)**	**P-value**
		Treatment session 7 (approx. 84 weeks)	3.8 (3.3)	NR	**–0.7**	NR
		Treatment session 8 (approx. 96 weeks)	3.8 (3.3)	NR	**–0.2**	NR
Lim 2008	OnabotA (Per-protocol analysis)	2 weeks	7.5	**5.9**	NR	NR
		6 weeks		**4.9**	NR	NR
		12 weeks		**3.2**	NR	p=0.064 (between groups)
	Triamcinolone acetonide (Per-protocol analysis)	2 weeks	7.6	**6.0**	NR	NR
		6 weeks		**5.5**	NR	NR
		12 weeks		**5.2**	NR	Reported for onabotA group
	OnabotA (Intention-to-Treat)	12 weeks	7.9	NR	**4.2**	p=0.051 (between groups)
	Triamcinolone acetonide (Intention-to-Treat)	12 weeks	7.6	NR	**2.5**	Reported for onabotA group
Restivo 2003	Overall cohort	8 days	2.6 (0.5)	**1.2 (0.8)**	NR	p<0.01 (pre vs post)
		30 days		**0.6 (0.5)**	NR	p<0.01 (pre vs post)
		90 days		**1.4 (1.3)**	NR	p<0.01 (pre vs post)
		120 days		**2.4 (0.5)**	NR	NR
Rousseaux 2002	Overall cohort	15 days	0.31 (0.36)	**0.24 (0.28)**	**–0.07 (0.14)**	NR
		2 months		**0.28 (0.32)**	**–0.03 (0.15)**	NR
		5 months		**0.19 (0.29)**	**–0.12 (0.21)**	NR
Wang 2002	Overall cohort	2 weeks	3.1*	**0.8***	NR	p≤0.05 (pre- vs post-intervention)
		4 weeks		**0.3***	NR	p≤0.05 (pre- vs post-intervention)
		8 weeks		**0***	NR	p≤0.05 (pre- vs post-intervention)

First author, year	Treatment cohort	Time-point of post-intervention measurement	Baseline mean value (SD)	Post-intervention value, mean (SD)	Change from baseline, mean (SD)	P-value
		12 weeks		**1.0***	NR	p≤0.05 (pre- vs post-intervention)
Wissel 2016	OnabotA + SC	12 weeks	3.4 (3.1)	NR	**-0.77 (95% CI: 1.14, 0.40)**	0.019
		24 weeks		NR	**-0.78 (95% CI: 1.22, 0.34)**	0.043
		52 weeks		NR	**-1.08 (95% CI: 1.52 to 0.65)**	NR
	Placebo + SC	12 weeks	3.7 (3.0)	NR	**-0.13 (95% CI: 0.51, 0.24)**	Reported for onabotA arm
		24 weeks		NR	**-0.13 (95% CI: 0.58, 0.31)**	Reported for onabotA arm
		52 weeks		NR	**-0.67, (95% CI: 1.12 to 0.22)**	NR

*Estimate from figure. All post-intervention values that are lower than baseline are **highlighted in bold.** CI, confidence interval; NR, not reported; SC, standard care; SD, standard deviation

 https://doi.org/10.3390/toxins17080418

Table S6. Newcastle-Ottawa Scale – Assessment of included observational studies

**Record**	**Selection**				**Comparability**	**Outcome**			**Overall score**
	Representativeness of the exposed cohort	Selection of the non-exposed cohort	Ascertainment of exposure	Demonstration that outcome of interest was not present at start of study	Comparability of cohorts on the basis of the design or analysis*	Assessment of outcome	Was follow-up long enough for outcomes to occur?	Adequacy of follow-up of cohorts	
**Bergfeldt 2006**		NA	★		NA		★	★	3
**De lcco 2019**		NA	★	★	★		★	★	5
**ASPIRE study:**	★	NA	★	★	NA		★	★	5
**Esquenazi 2021**	★	NA	★	★	NA		★		4
**Francisco 2020**	★	NA	★	★	NA		★	★	5
**Gordon 2004**		NA	★	★			★	★	4
**Jog 2016**	★	NA	★	★	★				4
**Marciniak 2008**		NA	★		NA				1
**Miscio 2004**		★	★	★	NA			★	4
**Reiter 1996**		NA	★	★	★	★	★		5
**Restivo 2003**		NA	★	★	NA		★	★	4
**Rousseaux 2002**		NA	★	★	NA		★	★	4
**Rousseaux 2014**		NA	★		NA		★	★	3

 https://doi.org/10.3390/toxins17080418

**Sampaio 1997**		NA	★	★	NA				2
**Slawek 2005**			★		NA		★	★	3
**Suputtitada 2002**		NA	★	★	NA		★	★	4
**Turhanoglu 2002**		NA	★	★	NA		★	★	4
**Wang 2002**		NA	★	★	NA		★	★	4
**Wissel 2000**	★	NA	★	★	NA		★	★	5

★ one score for the selection and outcome domain, up to two scores for the comparability domain. *Assessment on comparability of cohorts was non-applicable in studies with one treatment cohort which examined pain outcomes post-intervention versus baseline.
NA, not applicable

Disclaimer/Publisher's Note: The statements, opinions and data contained in all publications are solely those of the individual author(s) and contributor(s) and not of MDPI and/or the editor(s). MDPI and/or the editor(s) disclaim responsibility for any injury to people or property resulting from any ideas, methods, instructions or products referred to in the content.
